# Supplementary material for: Identification of photoperiod-induced specific miRNAs in the adrenal glands of Sunite sheep (Ovis aries)
Source: Front Vet Sci. 2022 Jul 22;9:888207. doi: 10.3389/fvets.2022.888207 (PMC9354845; doi:10.3389/fvets.2022.888207)
Supplement: Supplementary file 1 [file Data_Sheet_1.ZIP › Supplementary file/miRDeep2 file.zip!/miRDeep2 file/Know_Structure/LP42A1/oar-mir-30c.pdf]

miRBase precursor : oar-mir-30c
Total read count : 105484
oar-miR-30c read count : 105287
remaining reads : 197

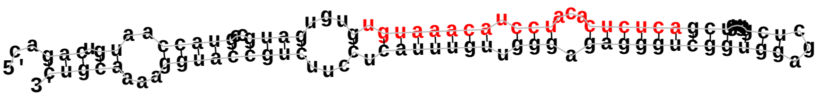

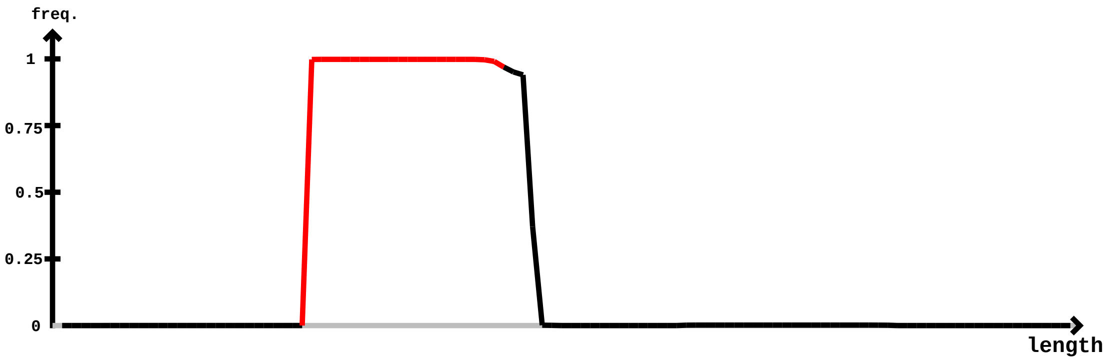


```
                                           oar-miR-30c
5'- cagacuguaaccaugccguagugugguguaaacauccuacacucucagcugugagcucgagguggcugggagaggguuguuuacuccuucugccauggaaaacguc  -3'   exp
    ..(((.((..(((((..((((...(.(((((((.(((...((((((((....(((...))))))))))))).))).))))))).)...)))))))))...)))))  reads  mm  sample
    cagacuguaaccaugccguagugu.................................................................................  1      0   seq
    cagacuguaaccaugccguagugug................................................................................  1      0   seq
    ..gacuguaaccaugccguagugug................................................................................  1      0   seq
    ....cuguaaccaugccguagugug................................................................................  3      0   seq
    ........................guguaaacauccuacacucucagc.........................................................  2      0   seq
    .........................uguaaacauccuacacu...............................................................  2      0   seq
    .........................uguaaacauccuacacuc..............................................................  127    0   seq
    .........................uguaaacaCccuacacuc..............................................................  1      1   seq
    .........................uguaaacauccuacacuU..............................................................  38     1   seq
    .........................uguaaacaCccuacacucu.............................................................  64     1   seq
    .........................uguGaacauccuacacucu.............................................................  4      1   seq
    .........................uguaaacauccuacacucu.............................................................  582    0   seq
    .........................uguaaacauccuacacuUu.............................................................  4      1   seq
    .........................uguaaacauccuacacucuc............................................................  2068   0   seq
    .........................uguaaacaCccuacacucuc............................................................  159    1   seq
    .........................uguaaacauccuacacucuca...........................................................  1776   0   seq
    .........................uguaaacaCccuacacucuca...........................................................  143    1   seq
    .........................uguaaacauccuacacucucag..........................................................  1059   0   seq
    .........................uguaaacaCccuacacucucag..........................................................  102    1   seq
    .........................uguaaacauccuacacucucagc.........................................................  55496  0   seq
    .........................uguaaacaCccuacacucucagc.........................................................  4546   1   seq
    .........................uguaaacaCccuacacucucagcu........................................................  2433   1   seq
    .........................uguaaacauccuacacucucagcu........................................................  36502  0   seq
    .........................uguaaacauccuacacucucagcug.......................................................  55     0   seq
    .........................uguaaacaCccuacacucucagcug.......................................................  3      1   seq
    .........................uguaaacauccuacacucucagcugu......................................................  83     0   seq
    .........................uguaaacaCccuacacucucagcugu......................................................  2      1   seq
    .........................uguaaacaCccuacacucucagcugug.....................................................  1      1   seq
    .........................uguaaacauccuacacucucagcugugagcu.................................................  1      0   seq
    ..........................guaaacauccuacacucuc............................................................  1      0   seq
    ..........................guaaacaCccuacacucucagc.........................................................  2      1   seq
    ..........................guaaacauccuacacucucagc.........................................................  21     0   seq
    ..........................guaaacauccuacacucucagcu........................................................  12     0   seq
    ................................................................cugggagaggguuguuuac......................  2      0   seq
    ................................................................cugggagaggguuguuuacu.....................  17     0   seq
```

oar-miR-30c

```
cagacuguaaccaugccguagugugUGUAAACAUCCUACACUCUCAgcugugagcucgagguggcugggagaggguuguuuacuccuucugccauggaaaacguc
................................................................cugggagaggguuguuuacuc....................    25    0    seq
................................................................cugggagaggguuguuuacucc...................    114   0    seq
................................................................cugggagaggguuguuuacuccu..................    2     0    seq
................................................................cugggagaggguuguuuacuccuu.................    1     0    seq
.................................................................ugggagaggguuguuuacu.....................    3     0    seq
.................................................................ugggagaggguuguuuacuc....................    3     0    seq
.................................................................ugggagaggguuguuuacucc...................    22    0    seq
```
